# Pharmacokinetics of 10-Hydroxy Mesaconitine in Rat Plasma by Ultra-Performance Liquid Chromatography-Tandem Quadrupole Mass Spectrometry

**DOI:** 10.1155/2021/6640184

**Published:** 2021-04-19

**Authors:** Jinzhao Yang, Guowu Zeng, Jianshe Ma, Xianqin Wang, Quan Zhou

**Affiliations:** ^1^Department of Pharmacy, The Third Affiliated Hospital of Shanghai University (Wenzhou People's Hospital), Wenzhou 325000, China; ^2^School of Pharmaceutical Sciences, Wenzhou Medical University, Wenzhou 325035, China; ^3^The Laboratory of Clinical Pharmacy, The People's Hospital of Lishui, Lishui 323000, China

## Abstract

Mesaconitine is the predominant active ingredient in *Aconitum carmichaelii* Debx. The compound 10-hydroxy mesaconitine is one known metabolite of mesaconitine and is toxic. In order to better understand its pharmacokinetics, UPLC-MS/MS was used in this paper to measure the concentration of 10-hydroxy mesaconitine in the plasma of rats after oral (5 mg/kg) and intravenous (0.1 mg/kg) administration of 10-hydroxy mesaconitine. The concentrations of 10-hydroxy mesaconitine in rat plasma measured in the standard curve covered the range of 0.3–60 ng/mL. The intraday and interday precisions of the samples of 10-hydroxy mesaconitine in rat plasma were lower than 15%. In addition, the accuracies ranged between 96.0% and 109.3%, the matrix effects ranged between 88.9% and 98.1%, and the recoveries were all higher than 79.1%. The AUC_(0 − *t*)_ values were 23.6 ± 5.9 and 207.6 ± 72.9 ng/mL·h for intravenous and oral administration, respectively, and the bioavailability of 10-hydroxy mesaconitine was 17.6%. Lastly, *t*_1/2_ was 1.3 ± 0.6 h and 3.1 ± 0.4 h for intravenous and oral administration, respectively.

## 1. Introduction

*Aconitum*, a genus of flowering plant species, is widely used in the clinic owing to its significant therapeutic effect in the treatment of rheumatic arthralgia [1–3]. *Aconitum* contains alkaloids such as mesaconitine and aconitine that exhibit anti-inflammatory and analgesic activity but at the same time suffer from strong toxicity and narrow therapeutic window [4–7]. In *Aconitum*, the amount of mesaconitine is higher than that of aconitine [4, 5]. *Aconitum* poisoning has no specific clinical symptoms because it rapidly decomposes in the body, making it difficult to detect [6, 7]. Metabolites of mesaconitine were detected in rat blood using liquid chromatography with tandem mass spectrometry (LC-MS/MS) [5], e.g., 10-hydroxyl-mesaconitine, hypaconitine, dehydrated mesaconitine 16-O-demethylmesaconitine, 16-O-demethylhypaconitine, and 16-O-demethyl-dehydrated hypaconitine. The metabolites of mesaconitine in rat urine were analyzed using LC-MS/MS [8], such as hypo-mesaconitine glucuronic acid conjugate, 10-hydroxy-mesaconitine, 1-O-demethyl mesaconitine, deoxy-mesaconitine, and hypo-mesaconitine. The metabolite 10-hydroxyl-mesaconitine is toxic and can be detected in both rat blood and urine; therefore, it was selected for quantification in this study.

LC-MS/MS is widely used in the detection and analysis of drugs [9–11], pharmaceutical impurities [12, 13], degradation products [14, 15], and metabolites [16, 17] because it has the advantages of high sensitivity, low detection limit, strong anti-interference ability, and accurate qualitative and quantitative measurability. Specifically, ultrahigh-performance liquid chromatography (UPLC) and a reliable internal standard can be used to reduce the effect of matrix effect on quantitative results. In addition, UPLC can achieve rapid and efficient qualitative and quantitative analysis of each component, improve the detection sensitivity and accuracy, and reduce the measurement time.

Several studies have been reported on the pharmacokinetic analysis of mesaconitine *in vivo* using HPLC [18], LC-MS/MS [19], and UPLC-MS/MS [20]. However, the pharmacokinetic analysis of 10-hydroxy mesaconitine *in vivo* has not been reported. In this study, UPLC-MS/MS was used to analyze the metabolite 10-hydroxy mesaconitine in rat plasma after oral and intravenous administration. This study is useful for elucidating the pharmacological effects of aconitine alkaloids.

## 2. Materials and Methods

### 2.1. Chemical

10-hydroxy mesaconitine (purity >98%) and diazepam (purity >98%, internal standard) were purchased from Chengdu Mansite Biotechnology Co., Ltd. (Chengdu, China). HPLC-grade formic acid, methanol, and acetonitrile were purchased from Tedia Company, Inc. (Ohio, USA). Ultrapure water was prepared using a Millipore Milli-Q water purification system (Bedford, MA, USA).

### 2.2. Instrument and Condition

An ACQUITY H-Class UPLC coupled with a XEVO TQS-micro triple quadrupole mass spectrometer was used in this study for the chromatographic analysis (Waters Corporation, Milford, MA, USA). A UPLC BEH C18 (2.1 mm × 50 mm, 1.7 *μ*m) column was used for chromatographic separation, and the mobile phase consisted of methanol and water (with 0.1% formic acid) with a flow rate of 0.5 mL/min. From 0 to 0.2 min, methanol was isocratic at 10%; from 0.2 min to 1.4 min, methanol was increased from 10% to 80%; from 1.4 min to 2.0 min, methanol was isocratic at 80%; from 2.0 min to 2.1 min, methanol was decreased from 80% to 10%; and from 2.1 min to 3.0 min, methanol was isocratic at 10%.

**Figure 1:**
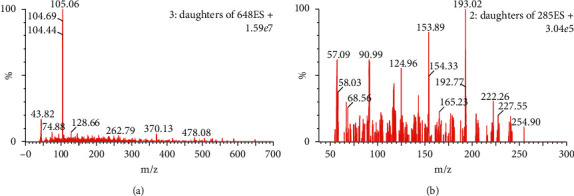
Mass spectrum of 10-hydroxy mesaconitine (a) and IS (b).

The capillary voltage was set to 2.4 kV, the desolvation temperature was 400°C, and the ion-source temperature was 150°C. Nitrogen was used as the desolvation (800 L/h) and nebulizing gas. Positive-ion mode ESI with multiple reaction monitoring (MRM) was used to monitor the molecular ion transitions of m/z 648 ⟶ 105 for 10-hydroxy mesaconitine and m/z 285 ⟶ 193 for the internal standard (IS) (Figure 1).

### 2.3. Preparation of Stock and Working Solutions

Stock solutions of 10-hydroxy mesaconitine (0.6 mg/mL) and diazepam (0.2 mg/mL) were prepared in methanol. Working stock solutions of 10-hydroxy mesaconitine at different concentrations (3, 10, 50, 150, 300, and 600 ng/mL) were prepared by diluting the stock solutions with methanol.

### 2.4. Preparation of the Standard Curve

Blank rat plasma was spiked with the working solutions of 10-hydroxy mesaconitine prepared previously to yield standard solutions of 10-hydroxy mesaconitine with concentrations of 0.3, 1, 5, 15, 30, and 60 ng/mL in plasma. Quality control samples (0.8, 12, and 50 ng/mL) were also prepared in the same manner.

### 2.5. Plasma Preparation

In a 1.5 mL centrifuge tube, acetonitrile (200 *μ*L, containing 50 ng/mL of the IS) was added to plasma (50 *μ*L). The solution was vortexed for 1 min and centrifuged at 13,000 rpm for 10 min. The resulting supernatant (150 *μ*L) was transferred to an LC-MS vial, and 2 *μ*L of this solution was injected into the UPLC-MS/MS for analysis.

### 2.6. Pharmacokinetics

Sprague Dawley (SD) rats (male, weight: 200–220 g) were obtained from the Animal Experimental Center of Wenzhou Medical University. Six rats were administered 5 mg/kg of 10-hydroxy mesaconitine orally (po), and six rats were administered 0.1 mg/kg of 10-hydroxy mesaconitine intravenously (iv). Food was prohibited for a period of 12 h before the start of the experiment, but water was freely available. Blood (150 *μ*L) was withdrawn from the caudal vein 0.083, 0.25, 0.5, 1, 2, 4, 6, 8, 12, and 24 h after administration of 10-hydroxy mesaconitine and was anticoagulated by heparin sodium. Plasma (50 *μ*L) was collected after centrifugation at 3,000 rpm for 15 min and stored at –20°C.

## 3. Results

### 3.1. Method Validation

**Figure 2:**
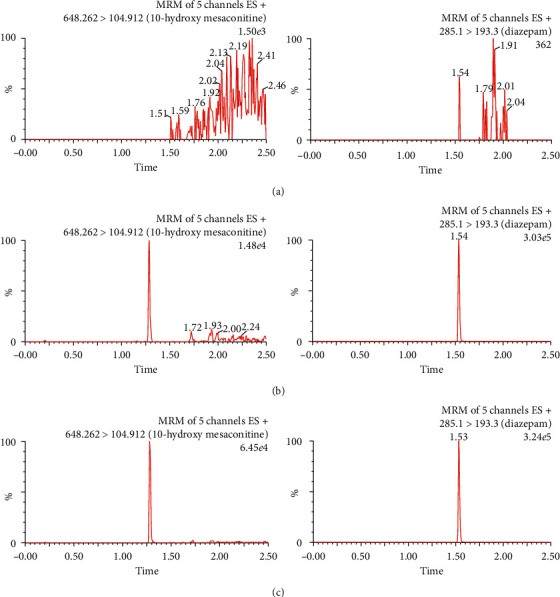
UPLC-MS/MS chromatograms of 10-hydroxy mesaconitine and IS in rat plasma. (a) Blank rat plasma. (b) Blank rat plasma spiked with 10-hydroxy mesaconitine and IS. (c) Rat plasma after oral administration of 10-hydroxy mesaconitine.

The selectivity of the method was evaluated by analyzing six lots of blank rat plasma, blank plasma spiked with 10-hydroxy mesaconitine and IS, and a rat plasma sample.  Figure 2 illustrates the UPLC-MS/MS chromatograms of blank plasma samples spiked with 10-hydroxy mesaconitine and IS. No endogenous substances were found to intervene in the detection of 10-hydroxy mesaconitine.

To assess the linearity, calibration curves were obtained by analyzing the spiked calibration samples (0.3–60 ng/mL in rat plasma) and plotting the peak area ratios of 10-hydroxy mesaconitine to IS against their concentrations. The concentrations of the standard 10-hydroxy mesaconitine solutions in rat plasma that were measured in the standard curve covered the range of 0.3–60 ng/mL. The equation generated from the standard curve was *y* = 0.0154*x* + 0.0124 (*r* = 0.9985), where *y* is the peak area ratio of 10-hydroxy mesaconitine to IS and *x* represents the 10-hydroxy mesaconitine concentrations in the rat plasma. The LLOQ of 10-hydroxy mesaconitine in rat plasma was determined to be 0.3 ng/mL.

The accuracy and precision were evaluated by the determination of the LLOQ and QC samples (0.8, 12, and 50 ng/mL) in six replicates over three consecutive days. Intraday and interday precisions corresponding to the measurement of 10-hydroxy mesaconitine in rat plasma were lower than 15%, and the accuracies ranged between 96.0% and 109.3%.

To evaluate the matrix effect, blank rat plasma was extracted and spiked with the 10-hydroxy mesaconitine to obtain final concentrations of 0.8, 12, and 50 ng/mL of the alkaloid. The matrix effect was determined by comparing the corresponding peak areas of the three quality control samples to those of standard solutions prepared in 1 : 1 methanol and water (0.1% formic acid) at equivalent concentrations. The recovery of 10-hydroxy mesaconitine was evaluated by comparing the peak areas of the extracted QC samples to those of the reference QC solutions. From the analysis, the matrix effect ranged between 88.9% and 98.1%, and the recoveries were higher than 79.1% (Table 1).

The stability of 10-hydroxy mesaconitine in rat plasma was evaluated by analyzing the peak areas of the three QC samples (0.8, 12, and 50 ng/mL) in rat plasma after being exposed to three different storage conditions: room temperature for 2 h; three freeze-thaw cycles, followed by room temperature for 12 h (after processing); and –20°C for 30 days. These results were compared to the results of the freshly prepared standard samples. The variation in the stabilities of 10-hydroxy mesaconitine in rat plasma for the three different conditions was within ±14%, and the RSD of these measurements was within ±14% (Table 2).

### 3.2. Pharmacokinetics

**Figure 3:**
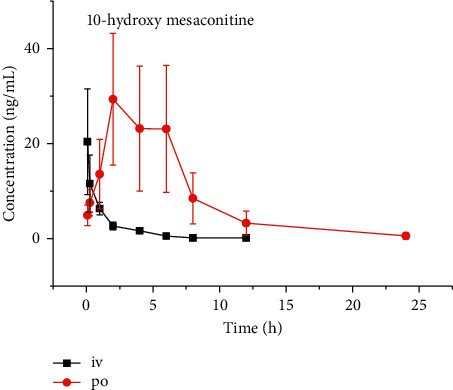
Plasma concentration-time curve of 10-hydroxy mesaconitine after oral (5 mg/kg) and intravenous (0.1 mg/kg) administration.

The plasma concentration-time curves of 10-hydroxy mesaconitine are shown in Figure 3. The main pharmacokinetic parameters determined by the non-compartment model using the DAS 2.0 software (China Pharmaceutical University) are listed in Table 3.

## 4. Discussion

The choice of operating in either positive or negative mode for ESI is often evaluated in mass spectrometry methodologies [21, 22]. It was determined that ESI positive-ion mode was more suitable for the mass spectrometry analysis of 10-hydroxy mesaconitine compared to negative-ion mode. We optimized the ionization of 10-hydroxy mesaconitine and found that the fragment with an m/z ratio of 105 had the highest abundance. Therefore, the transition of m/z 648 ⟶ 105 for 10-hydroxy mesaconitine (cone voltage: 35 V; fragmentor voltage: 26 V) was monitored for quantitative analysis.

The mobile phase and stationary phase (column) play a decisive role in the chromatographic behavior [23–25]. We compared methanol-water (w/0.1% formic acid) and acetonitrile-water (w/0.1% formic acid) using BEH C18 columns. A suitable retention time and analyte peak shapes could be obtained by using methanol-water (w/0.1% formic acid) as the mobile phase. Compared to acetonitrile, methanol was more appropriate as the organic phase for a suitable retention of 10-hydroxy mesaconitine on the column, and it produced better peak shapes and sensitivity. UPLC can obtain excellent results by enabling the efficient separation of analytes with a wide linear velocity, flow rate, and back pressure. UPLC manifests improved chromatographic resolutions compared to HPLC by offering advantages that range from smaller (1.7 *μ*m) particles to an increased column efficiency. In particular, the column efficiency provided by the 1.7 *μ*m particles is three times higher compared to the efficiency of columns containing 5 *μ*m particles. Using methanol-water (w/0.1% formic acid) as the mobile phase, the retention times of 10-hydroxy mesaconitine and the IS were 1.28 and 1.54 min, respectively.

Eliminating potential interferences is especially important for sample preparation [26–28]. The use of methanol-acetonitrile (1 : 1, v/v), acetonitrile, and methanol for precipitation protein was investigated. Acetonitrile seemed to be the best choice because it afforded better extraction recoveries and a lower background interference. The extraction recoveries with ethyl acetate and ethyl ether were about 60%, while the extraction recoveries with methanol and methanol-acetonitrile (1 : 1, v/v) were about 80%. However, the recovery with acetonitrile was about 90%.

In this experiment, a proprietary UPLC-MS/MS method was established to determine the concentration of 10-hydroxy mesaconitine in rat plasma. This method was sensitive, accurate, specific, and reliable. The analysis time for each sample was 3.0 min, the processing method of the plasma sample was trivial, the required sample volume was only 50 *μ*L, and more than 300 plasma samples could be processed and tested every day. The developed UPLC-MS/MS method was applied in the pharmacokinetic analysis of 10-hydroxy mesaconitine in rats after oral (5 mg/kg) and intravenous (0.1 mg/kg) administration. The AUC_(0 − *t*)_ values were 23.6 ± 5.9 and 207.6 ± 72.9 ng/mL·h for intravenous and oral administration, respectively, and the bioavailability was 17.6%. *t*_1/2_ was 1.3 ± 0.6 h and 3.1 ± 0.4 h for intravenous and oral administration, respectively.

## 5. Conclusion

*Aconitum* alkaloids get metabolized rapidly *in vivo*, which makes them difficult to detect. In this study, a rapid and selective UPLC-MS/MS method for the measurement of 10-hydroxy mesaconitine in rat plasma was developed. This method was applied in the pharmacokinetic analysis of 10-hydroxy mesaconitine in rats. The bioavailability was 17.6%.

## Figures and Tables

**Table 1:** Accuracy, precision, matrix effect, and recovery of 10-hydroxy mesaconitine in rat plasma.

Concentration (ng/mL)	Accuracy (%)		Precision (%RSD)		Matrix effect (%)	Recovery (%)
	Intraday	Interday	Intraday	Interday		
0.3	107.9	109.3	11.8	15.0	88.9 ± 7.8	93.0 ± 6.7
0.8	103.1	103.2	9.2	8.3	94.2 ± 6.5	79.1 ± 5.3
12	98.1	94.3	6.8	11.3	96.5 ± 4.5	87.7 ± 3.2
50	97.2	102.1	4.5	6.1	98.1 ± 5.6	92.2 ± 3.4

**Table 2:** Summary of the stability of 10-hydroxy mesaconitine under various storage conditions (*n* = 3).

Concentration (ng/mL)	Autosampler (4°C, 12 h)		Ambient (2 h)		–20°C (30 d)		Freeze-thaw	
	Accuracy (%)	RSD (%)	Accuracy (%)	RSD (%)	Accuracy (%)	RSD (%)	Accuracy (%)	RSD (%)
0.8	97.2	1.8	102.9	8.0	112.7	9.0	89.9	12.4
12	97.5	6.7	109.1	6.0	95.6	9.7	101.4	8.9
50	103.0	0.5	94.5	2.1	97.0	10.4	86.7	10.6

**Table 3:** Main pharmacokinetic parameters of 10-hydroxy mesaconitine in rats.

Parameters	Unit	iv 0.1 mg/kg	po 5 mg/kg
AUC_(0 − *t*)_	ng/mL·h	23.6 ± 5.9	207.6 ± 72.9
AUC_(0 − ∞)_	ng/mL·h	23.7 ± 5.9	208.9 ± 73.1
*T*_1/2*z*_	h	1.3 ± 0.6	3.1 ± 0.4
*V*_*z*_	L/kg	0.083	
CL_*z*_	L/h/kg	8.1 ± 4.4	
*V*_z_/*F*	L/kg		2.8 ± 1.8
CL_*z*_/*F*	L/h/kg		117.9 ± 47.3
*C*_max_	ng/mL	4.4 ± 1.0	26.3 ± 8.6
Bioavailability			17.6%

## Data Availability

The data used to support the findings of this study are included within the article.

## References

[1] Tan Y., Liu X., Lu C. (2014). Metabolic profiling reveals therapeutic biomarkers of processed Aconitum Carmichaeli Debx in treating hydrocortisone induced Kidney-Yang deficiency syndrome rats. *Journal of Ethnopharmacology*.

[2] Tong P., Xu S., Cao G. (2014). Chondroprotective activity of a detoxicated traditional Chinese medicine (Fuzi) of Aconitum carmichaeli Debx against severe-stage osteoarthritis model induced by mono-iodoacetate. *Journal of Ethnopharmacology*.

[3] Wang D.-P., Lou H.-Y., Huang L. (2012). A novel franchetine type norditerpenoid isolated from the roots of Aconitum carmichaeli Debx. with potential analgesic activity and less toxicity. *Bioorganic & Medicinal Chemistry Letters*.

[4] Sun B., Zhang M., Zhang Q. (2014). Metabonomics study of the effects of pretreatment with glycyrrhetinic acid on mesaconitine-induced toxicity in rats. *Journal of Ethnopharmacology*.

[5] Tan P., Chen X., He R., Liu Y. (2014). Analysis of the metabolites of mesaconitine in rat blood using ultrafast liquid chromatography and electrospray ionization mass spectrometry. *Pharmacognosy Magazine*.

[6] Lam R. P. K., Leung J. K. S., Tsui M. S. H. (2018). Clinical and electrocardiographic features of aconitine poisoning presenting to the emergency department. *Clinical Toxicology*.

[7] Hermanns-Clausen M., Katlein R., Ionascu I., Auwarter V. (2018). Analytically confirmed aconitine poisoning as a result of mistaking monkshood leaves for lovage. *Clinical Toxicology*.

[8] Chen P. P., Zhao N., Xu X. L., Ruan Y. P., Wei Y. H., Li F. Z. (2010). Analysis on the metabolites of mesaconitine in the rat urine by liquid chromatography and electrospray ionization mass spectrometry. *Yao Xue Xue Bao*.

[9] Zhu X., Huo S., Xue C., An B., Qu J. (2020). Current LC-MS-based strategies for characterization and quantification of antibody-drug conjugates. *Journal of Pharmaceutical Analysis*.

[10] Veach B. T., Bandara N., Broadaway B. (2020). Determination of chloramphenicol and nitrofuran metabolites in cobia, croaker, and shrimp using microwave-assisted derivatization, automated SPE, and LC-MS/MS-Results from a U.S. food and drug administration level three inter-laboratory study. *Journal of Aoac International*.

[11] Barco S., Castagnola E., Mesini A., Tripodi G., Cangemi G. (2019). Potential pitfalls in LC-MS/MS quantification of colistin for therapeutic drug monitoring of patients treated with colistimethate. *Journal of Pharmaceutical and Biomedical Analysis*.

[12] Kushwaha M., Goel B., Jaglan S., Jain S. K. (2020). LC-MS/MS profile of an active pharmaceutical ingredient and its impurities in commercial preparation. *Journal of Liquid Chromatography & Related Technologies*.

[13] Antolcic M., Runje M., Galic N. (2020). A simple and sensitive LC-MS/MS method for determination and quantification of potential genotoxic impurities in the ceritinib active pharmaceutical ingredient. *Analytical Methods*.

[14] Ragi N. C., Velma G. R., Pallerla P. K. (2020). Identification and characterization of forced degradation products of vortioxetine by LC/MS/MS and NMR. *Journal of Pharmaceutical and Biomedical Analysis*.

[15] Özcan S., Levent S., Geven A., Özkay Y., Can N. Ö. (2020). Stability-indicating LC-MS/MS and LC-DAD methods for robust determination of tasimelteon and high resolution mass spectrometric identification of a novel degradation product. *Journal of Pharmaceutical and Biomedical Analysis*.

[16] Takenaka M., Yoshida T., Hori Y. (2021). An ion-pair free LC-MS/MS method for quantitative metabolite profiling of microbial bioproduction systems. *Talanta*.

[17] Rukthong P., Sereesongsang N., Kulsirirat T., Boonnak N., Sathirakul K. (2020). In vitro investigation of metabolic fate of alpha-mangostin and gartanin via skin permeation by LC-MS/MS and in silico evaluation of the metabolites by ADMET predictor (TM). *Bmc Complementary Medicine and Therapies*.

[18] Yang Y., Chen J., Shi Y.-P. (2010). Determination of aconitine, hypaconitine and mesaconitine in urine using hollow fiber liquid-phase microextraction combined with high-performance liquid chromatography. *Journal of Chromatography B*.

[19] Li M., Cai Z. (2013). Simultaneous determination of aconitine, mesaconitine, hypaconitine, bulleyaconitine and lappaconitine in human urine by liquid chromatography-electrospray ionization-tandem mass spectrometry. *Analytical Methods*.

[20] Ye L., Gao S., Feng Q. (2012). Development and validation of a highly sensitive UPLC-MS/MS method for simultaneous determination of aconitine, mesaconitine, hypaconitine, and five of their metabolites in rat blood and its application to a pharmacokinetics study of aconitine, mesaconitine, and hypaconitine. *Xenobiotica*.

[21] Zhou Q., Zhang Z., Geng P., Huang B., Wang X., Yu X. (2020). Pharmacokinetics of ligustroflavone in rats and tissue distribution in mice by UPLC-MS/MS. *Acta Chromatographica*.

[22] Xie H., Lu X., Jin W. (2020). Pharmacokinetics of picroside I, II, III, IV in rat plasma by UPLCMS/MS. *Current Pharmaceutical Analysis*.

[23] Yao Y. X., Wang B., Lin Z. X. (2016). Tissue distribution and pharmacokinetics of chidamide based on UPLC-MS/MS. *Latin American Journal of Pharmacy*.

[24] Li J., Yu Z., Han C. (2020). Determination of diosmetin-7-o-*β*-d-glucoside in rat plasma by UPLC-MS/MS. *Acta Chromatographica*.

[25] Li J., Jin Y., Fu H., Huang Y., Wang X., Zhou Y. (2019). Pharmacokinetics and bioavailability of gelsenicine in mice by UPLC-MS/MS. *Biomedical Chromatography*.

[26] Song Y., Wang Z., Feng X., Deng X., Zhu J. (2016). Simultaneous determination and pharmacokinetics of four triterpenoids by ultra high performance liquid chromatography with tandem mass spectrometry after the oral administration of *Acanthopanax sessiliflorus* (Rupr. et Maxim) Seem extract. *Journal of Separation Science*.

[27] Lin G. T., Chen Y. Y., Yu Y., Wang H. Z., Wang X. Q., Chen L. M. (2020). Pharmacokinetics of talatisamine in rat plasma by UPLC-MS/MS. *Latin American Journal of Pharmacy*.

[28] Weng Q. H., Zhang Z. N., Chen L. L. (2020). Quantitative determination of ginsenoside Rg1 in rat plasma by ultrahigh performance liquid chromatography-tandem mass spectrometry (UHPLC-MS/MS) and its application in a pharmacokinetics and bioavailability study. *Current Pharmaceutical Analysis*.

